# Advancing the science of genomic learning healthcare systems

**DOI:** 10.1002/lrh2.70027

**Published:** 2025-07-23

**Authors:** Teri A. Manolio, Renee Rider, Carol J. Bult, Rex L. Chisholm, Patricia A. Deverka, Geoffrey S. Ginsburg, Eric D. Green, Gail P. Jarvik, George A. Mensah, Jahnavi Narula, Erin M. Ramos, Mary V. Relling, Dan M. Roden, Robb Rowley, Noura S. Abul‐Husn, Adam H. Buchanan, Christopher G. Chute, Guilherme Del Fiol, Gai Elhanan, Susanne B. Haga, Rizwan Hamid, Carol R. Horowitz, Peter J. Hulick, Cynthia A. James, Janina M. Jeff, Bruce Korf, Latrice Landry, Deven McGraw, Howard L. McLeod, Nancy J. Mendelsohn, Travis Osterman, Casey Overby Taylor, Daryl Pritchard, Heidi L. Rehm, Krystal S. Tsosie, Jason L. Vassy, Karriem Watson, Ken Wiley, Marc S. Williams

**Affiliations:** ^1^ National Human Genome Research Institute National Institutes of Health Bethesda Maryland USA; ^2^ The Jackson Laboratory for Mammalian Genetics Bar Harbor Maine USA; ^3^ Center for Genetic Medicine, Feinberg School of Medicine Northwestern University Chicago Illinois USA; ^4^ Deverka Consulting, LLC Durham North Carolina USA; ^5^ Center for Translational and Policy Research on Precision Medicine (TRANSPERS), Department of Clinical Pharmacy University of California San Francisco California USA; ^6^ All of Us Research Program National Institutes of Health Bethesda Maryland USA; ^7^ Department of Medicine (Medical Genetics) and Genome Sciences University of Washington Seattle Washington USA; ^8^ Center for Translation Research and Implementation Science, National Heart, Lung, and Blood Institute National Institutes of Health Bethesda Maryland USA; ^9^ Pharmaceutical Sciences Department St. Jude Children's Research Hospital Memphis Tennessee USA; ^10^ Department of Medicine, Pharmacology, and Biomedical Informatics Vanderbilt University Medical Center Nashville Tennessee USA; ^11^ Genomic Health 23andMe Sunnyvale California USA; ^12^ Division of Genomic Medicine, Department of Medicine, Institute for Genomic Health Icahn School of Medicine at Mount Sinai New York New York USA; ^13^ Department of Genomic Health Geisinger Danville Pennsylvania USA; ^14^ Schools of Medicine, Public Health and Nursing, and Institute for Clinical and Translational Research Johns Hopkins University Baltimore Maryland USA; ^15^ Department of Biomedical Informatics University of Utah Salt Lake City Utah USA; ^16^ School of Medicine University of Nevada Reno Nevada USA; ^17^ Division of General Internal Medicine, Department of Medicine Duke University School of Medicine Durham North Carolina USA; ^18^ Department of Pediatrics and Vanderbilt Center for Undiagnosed Diseases Vanderbilt University Medical Center Nashville Tennessee USA; ^19^ Department of Population Health Science and Policy and Institute for Health Equity Research Icahn School of Medicine at Mount Sinai New York New York USA; ^20^ Division of Medical Genetics and Neaman Center for Personalized Medicine Endeavor Health Evanston Illinois USA; ^21^ Department of Human Genetics University of Chicago Pritzker School of Medicine Chicago Illinois USA; ^22^ Department of Cardiology and Genetic Medicine Johns Hopkins University Baltimore Maryland USA; ^23^ Illumina San Diego California USA; ^24^ Department of Genetics University of Alabama at Birmingham Birmingham Alabama USA; ^25^ Department of Genetics, Perelman School of Medicine University of Pennsylvania Philadelphia Pennsylvania USA; ^26^ Data Stewardship and Data Sharing Citizen Health San Francisco California USA; ^27^ Center for Precision Medicine and Functional Genomics Utah Tech University St. George Utah USA; ^28^ NJMendelsohn Consulting Eden Prairie Minnesota USA; ^29^ Vanderbilt University Medical Center Nashville Tennessee USA; ^30^ Department of Medicine and Biomedical Engineering and Institute of Computational Medicine Johns Hopkins University Baltimore Maryland USA; ^31^ Personalized Medicine Coalition Washington DC USA; ^32^ Center for Genomic Medicine Harvard Medical School Boston Massachusetts USA; ^33^ Center for Genomic Medicine Massachusetts General Hospital Boston Massachusetts USA; ^34^ School of Life Sciences Arizona State University Tempe Arizona USA; ^35^ Division of General Internal Medicine and Primary Care Brigham and Women's Hospital Boston Massachusetts USA; ^36^ VA Center for Health Optimization and Implementation Research VA Boston Healthcare System Boston Massachusetts USA; ^37^ UIC School of Public Health and UI Health Mile Square Health Center Chicago Illinois USA; ^38^ Division of Clinical Innovation National Center for Advancing Translational Sciences Rockville Maryland USA

**Keywords:** genomic medicine, genomics, informatics

## Abstract

**Introduction:**

Identifying key characteristics of exemplar genomic learning healthcare systems (gLHS) and knowledge gaps that can be explored by collaboration among them is likely to accelerate the sharing of best practices and generation of evidence that informs the use of genomics in clinical care.

**Methods:**

Deliberations of an expert group convened by the National Human Genome Research Institute (NHGRI) supplemented by relevant literature.

**Results:**

Recent advances in genomic data standardization, automated clinical decision support, increased interoperability, and improved genomic technologies have enabled the development of several robust gLHS. They remain concentrated in major academic centers, however, and operate largely independently. Sharing their methods and tools would increase access to these innovations and advance the field. Several gLHS have expressed willingness to collaborate in a coalition designed to gather, evaluate, and disseminate best practices and development needs. Such a coalition has recently been formed under the leadership of NHGRI.

**Conclusion:**

Increased collaboration, interoperability, and sharing of genomic information and strategies across gLHS can help define, refine, and disseminate best practices. Such cooperation can improve genomic variant curation and interpretation, diagnostic accuracy, evidence generation, and ultimately patient care through seamless integration of research as an integral component of good clinical care.

## 1 INTRODUCTION

Learning healthcare systems (LHS) have been defined as those “in which science, informatics, incentives, and culture are aligned for continuous improvement and innovation… and new knowledge [is] captured as an integral by‐product of the care experience”.^1^ LHS involve iterative rounds of development and optimization for identifying new knowledge related to improvements in clinical care, implementing that knowledge within a healthcare system, collecting and analyzing data on the impact of the implementation, and using those findings to design and deploy improvements in a virtuous cycle of innovation and implementation.^1^, ^2^ Genomic information can be integrated at any step in this process to produce “genomic learning healthcare systems” (gLHS) that identify and implement new *genomic* knowledge and assess the resulting impact.^2^ gLHS can thus facilitate rapid incorporation and evaluation of genomics‐informed care, particularly if these systems collaborate to investigate research gaps and generate evidence of gLHS effectiveness.

gLHS have been made possible through rapid improvements in genomic technologies^2^ as well as the widespread availability of electronic health records (EHRs) and associated information technology, progress in implementation science, and patient/community engagement.^1^ Central to their realization are EHR systems integrated with large, distributed databases and thousands to millions of patient records.^3^ gLHS can facilitate the clinical use of the vast array of genomic information potentially available on an individual, promote appropriate (and reduce duplicative) genomic testing,^4^ and facilitate collaboration to improve care and support research.^3^ Barriers to realizing these opportunities, however, include the lack of transportability of genomic data and interoperability of current data systems, fueled by limited use of data standards and lack of scalable clinical decision support (CDS), as well as limited genomic variation in gLHS databases or agreed‐upon protections for data privacy.^3^ Common barriers to implementing genomics in clinical care (in addition to these primarily data‐focused issues), such as apprehension or lack of familiarity with genomics among patients and clinicians, lack of coverage and reimbursement, etc., pertain to implementing gLHS as well.

Several of these barriers are beginning to be addressed within individual gLHS.^5^, ^6^, ^7^ To explore successful implementation models and the potential for collaboration among them to address research gaps, the National Human Genome Research Institute (NHGRI) convened 30 experts in genomic medicine, clinical informatics, patient advocacy, health equity, and other disciplines in 2022 to review recent progress in addressing gLHS implementation challenges. Here we summarize these discussions, highlighting key characteristics and recent advances of exemplar gLHS as well as important research gaps, and identify ways to build upon and disseminate these advances.

## 2 CHARACTERISTICS OF OPTIMAL gLHS

Many of the characteristics described below as important to optimizing gLHS are similar to LHS in general but are complicated by the massive volume and persisting and evolving nature of genomic data. In contrast to virtually all other healthcare data, germline genomic data remain unchanged over the lifetime of a patient necessitating storage and access solutions that are not needed for other data types. While it is impractical and unnecessary to integrate all 3 billion base pairs into a patient's record, even filtering down to just the roughly 1 million variant sites is probably excessive. Limiting EHR integration to pathogenic and likely pathogenic (P/LP) variants along with pharmacogenetically significant variants would probably suffice, but variants of uncertain significance would need to be stored and periodically queried in the background and presented to the EHR when they reach the P/LP threshold. Systems presenting these data to clinicians, using them to affect management, and assessing the impact of that use must be capable of ingesting and computing on them and be context aware such that information is only presented at the appropriate time in the clinical decision‐making process. These systems also need the ability to update variant interpretation as genomic knowledge accrues. One proposed solution is creation of a so‐called “Omic Ancillary System” analogous to a picture archiving and communication system (PACS) used in radiology.^8^ Such an ancillary system has been implemented at Northwestern University to support the pharmacogenomic service.^9^

*Robust, clinician‐friendly, and patient‐centered data infrastructure*. Key features of a data infrastructure that enables gLHS were identified by a 2015 National Academy of Medicine Roundtable^3^ and are updated here (Box 1), including some that may only be aspirational at present. In addition to a robust data infrastructure capable of securely integrating multiple data types and sources and enabling real‐time analyses and feedback, gLHS data systems need to be clinician‐friendly and integrated into routine clinical workflows. The information they provide should be both useful and timely for patient care. A recent systematic review assessing the implementation of genomic Clinical Decision Support Systems (gCDS) noted very few implemented examples of clinician‐friendly data systems integrated into clinical workflows; the majority of the implemented gCDS was in a pilot project.^10^ Data infrastructures should also permit facile and bi‐directional information exchange among clinicians, patients, and testing laboratories to improve genomic variant interpretation, diagnostic accuracy, results reporting, and clinical management. These infrastructures should also be patient‐centered, easily accessible, and able to include outcomes and products that patients view as valuable to them. Other important perspectives of patients should also be captured, including whether and how genomic results will be returned to them and preferences for participating in research or sharing their data. To our knowledge there are as yet no fully implemented examples of systems with these features, although one solution, GeneInsight, was implemented across the electronic Medical Records and Genomics (eMERGE) Network to support the return of genomic results.^11^

**BOX 1:** Characteristics of data infrastructures and healthcare systems that enable gLHS (*aspirational, for future development).

Provide robust data infrastructure that is clinician‐ and patient‐friendly Integrate multiple diverse types and sources of information, including clinical data, genomic data, and laboratory data Facilitate NLP and other data extraction methods to operationalize algorithms for SDOH* Use widely accepted standards for these data types permitting interoperability and data sharing* Ensure data security and safeguarded access Provide application programming interfaces (APIs) and visualizations of findings in user‐friendly ways [Figure 2], potentially including multi‐omics data [Figure 3] that can be communicated at the point of care to users (both clinicians and patients) with limited knowledge of genomics Provide platform‐supported, rapid learning cycles that enable bidirectional analysis and feedback to produce results and use those results to change practices* Systematically and continually gather and apply evidence in real time to guide care Promote transferability of longitudinal health data* Speed clinician interpretation and use Integrate seamlessly into clinical workflow* Provide point‐of‐care clinical decision support and just‐in‐time guidelines for clinical action Enable options for additional information for interested users Promote active information exchange between clinician and laboratory to improve classification of genomic variants, interpretation of findings, and accuracy of diagnoses* Expand genomic medicine knowledge of non‐genetics clinicians* Enhance patient understanding and engagement Allow patients to define preferences about return of results, communication to family members, use and sharing of information* Include dynamic updates of clinical information and preferences* Include outcomes of interest to patients Incorporate ongoing monitoring, follow‐up, updating Use patient portals tethered to EHR to enhance patient engagement with their genomic information Put data in patients' hands so they can carry data and diagnoses from system to system as they move* Support use of genomics‐enabled EHRs Receive structured genomic test results in standardized formats directly into EHR, permitting background automated querying and alerting for actionable updates Capture and support both human‐viewable and machine‐readable formats Maintain linkage of molecular observations to laboratory methods used to generate them Support real‐time data access and rapid querying at level of individual patient and system‐wide Support facile integration of new genomic knowledge and clinical decision support* Enable research Utilize implementation frameworks to address barriers and facilitators for implementation systematically Capture the cost of gLHS and demonstrate its economic feasibility and benefits for healthcare system and patients* Standardize consent with multiple options for data sharing and research use* Ensure equity and minimize biases Provide equal, effective, affordable access to genomic medicine implementation* Ensure ongoing monitoring of equity and engagement of stakeholders from disadvantaged communities to refine and improve equity of access and use* Utilize genomic variant data in combination with self‐identified race/ethnicity to reduce biases in predicting risk, especially when confounding by health inequities can be measured and minimized* Gather community input for population descriptors to use besides self‐identified race/ethnicity and genomic variant data to reduce biases in predicting risk and to measure health inequities* Include participation of disadvantaged populations in gLHS research, not only as study participants but also as investigators and engaged communities Provide clinical and informatics solutions for under‐resourced settings Ensure meaningful, ongoing community engagement to ensure relevance and implementation of findings in communities at greatest need

**FIGURE 1:**
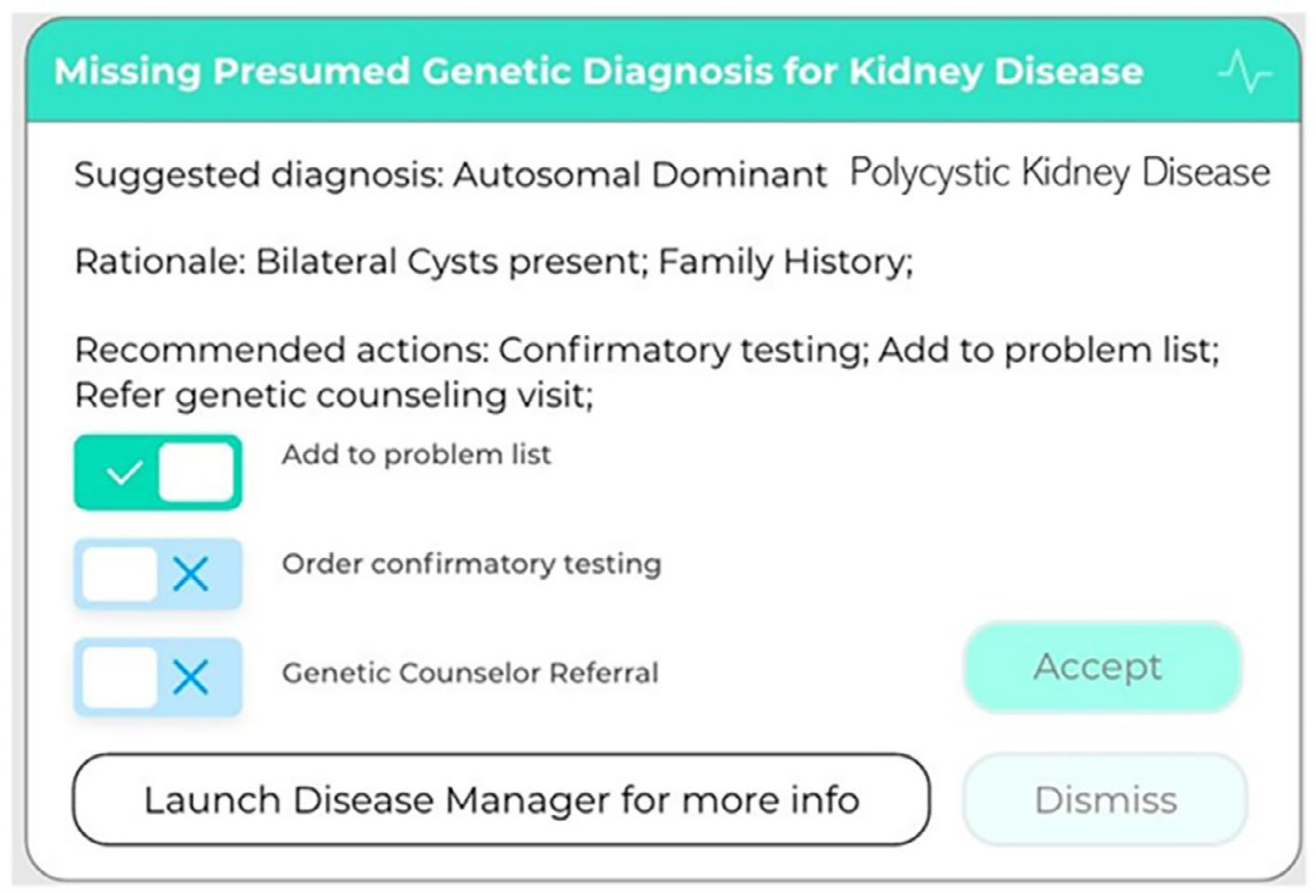
Example of EHR algorithm providing actionable information on possible genetic diagnosis of kidney disease at the point of care, with additional information available if desired. (M. Williams, personal communication).

*Research‐enabled EHRs*. Clinical care and research are inextricably bound in well‐functioning gLHS, so ensuring the integrity and availability of clinical data is critical. EHRs enabled to receive digital genomic results in a structured format (as opposed to the near‐universal current practice of sending only PDFs) facilitate ongoing, automated algorithmic queries as clinical conditions evolve and genomic knowledge accrues.^12^ Clinical care environments linked to genomic data within or accessible by EHRs can robustly support discovery and inform clinical workflows. Running such algorithms in the EHR “background” and providing information at the point‐of‐care only when it is actionable (Figure 1) optimize the clinical value of germline genome sequence data, which are essentially invariant throughout the lifespan (though their interpretation and clinical significance may change). Genomics‐enabled EHRs should also permit large‐scale analytics for clinical and research purposes as well as quality improvement studies that promote the virtuous cycle of innovation and implementation in gLHS. Thus, a key feature of such systems is the delivery and storage of genomic information in digital and readily retrievable formats.

**FIGURE 2:**
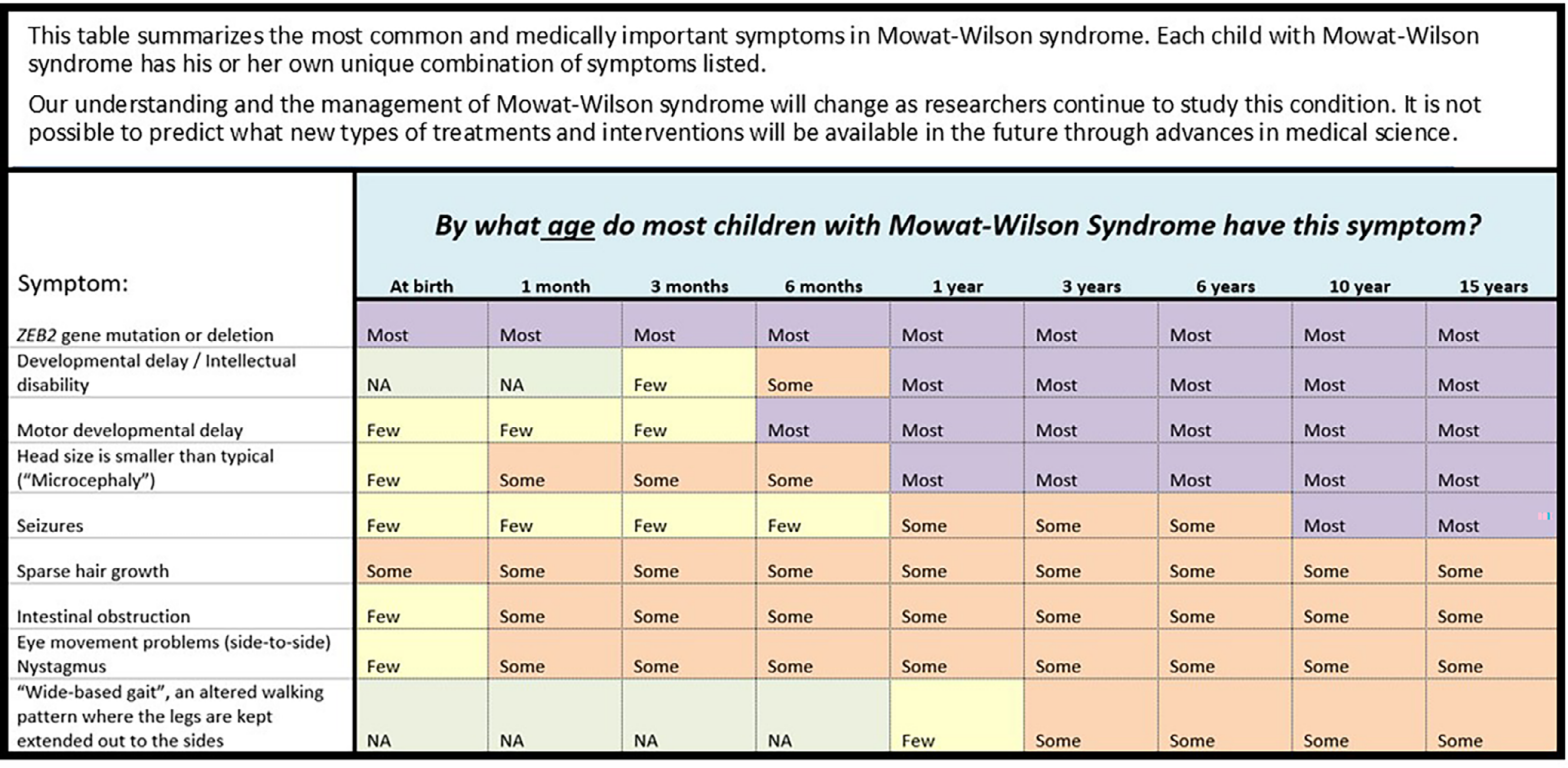
Example of genomic report visualization that can be communicated at the point of care to users (both clinicians and patients) with limited knowledge of genomics.^45^

**FIGURE 3:**
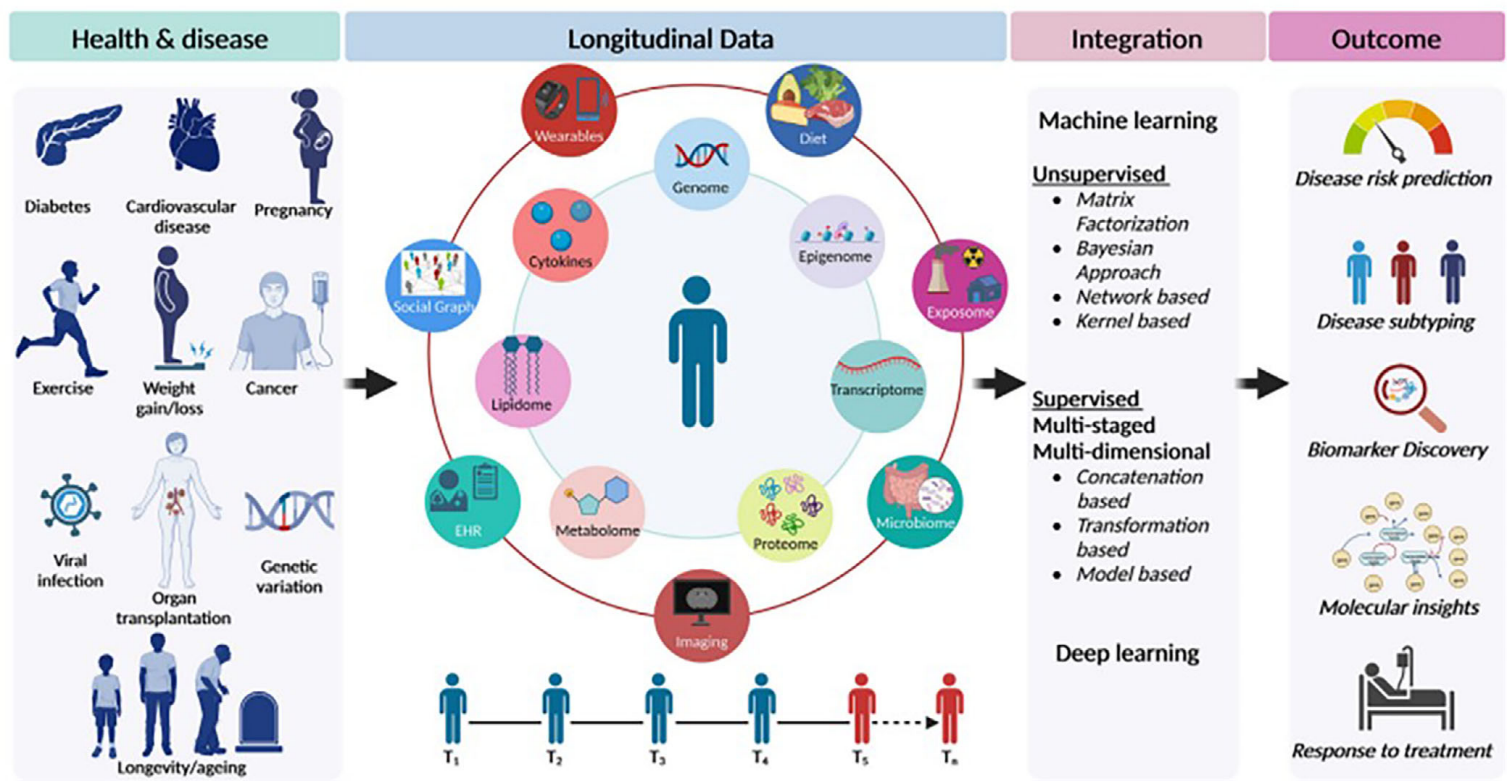
Longitudinal multi‐omics and wearable data enabled deep phenotyping for precision health. Omics and non‐omics data across times (T1–Tn) could be integrated using machine learning and deep learning approaches to predict disease risk, subtyping, biomarker discovery, molecular insights, and response to treatment, among others.^46^

*Equity and bias*. gLHS should equitably provide “effective and affordable access to genomic medicine… without bias for or against any group”.^13^ This is a tall order given the systematic over‐representation of European ancestry populations in genomics research, results of which may translate poorly to other populations.^13^ Current gLHS largely continue that over‐representation, both in who has access to clinical genetic testing and to genomic research. gLHS research should identify and reduce barriers to providing care to underrepresented (and often underserved) groups as well as facilitate their participation in genomic research. In this way, participation of diverse populations as study participants, investigators, and engaged communities may be substantially increased. gLHS must also work to assess and minimize biases analytically or by sampling or expanded data collection. Outcomes to assess the value of genomic medicine programs must not only be harmonized across programs^14^ but also be evaluated to ensure that these outcomes do not reflect inherent biases that would impede equitable application. Such biases can confound interpretation of genomic associations in disadvantaged populations, such as social determinants of health (SDOH) if there is co‐occurrence of specific variants with SDOH in these populations. Innovative natural language processes and other data extraction methods, such as emerging Large Language Models^15^ are needed to operationalize algorithms for SDOH interpretation and use in care.

*Under‐resourced settings*. The substantial informatics infrastructure needed to use genomic data efficiently is less likely to be available in under‐resourced settings, which disproportionately serve populations suffering health disparities, limiting the generalizability of gLHS approaches. Creative solutions to address these biases and to promote health equity could be explored by a gLHS network if this is defined as a priority. Such solutions are needed for implementing gLHS while not worsening disparities in health outcomes and segregation of gLHS‐informed care. Community engagement can help address health inequities but must go beyond simple consultation. Rather, intentional community engagement should include active, ongoing participation of community leaders, advocacy groups, front‐line clinicians, and patients to inform the design and dissemination of findings. Such efforts may promote inclusive participation, contribute novel questions and strategies, enhance the relevance of research findings, increase trust and acceptance of research and genomics‐enabled clinical care, and improve dissemination of scientific advances to communities and clinicians.^16^

## 3 MAJOR ADVANCES OF CURRENT gLHS

Integration of structured genomic test results into the EHR is now possible in over two dozen US healthcare systems [T Osterman, presentation]. Although most integration currently occurs through manual entry of selected genomic results, work is progressing on direct, automated data transfer from genomic testing labs.^5^ Integrated genomic results are then available for CDS to deliver recommendations seamlessly to the pointofcare.^6^ Achieving these end‐to‐end aims requires substantial institutional investment, multidisciplinary participation, clinical and educational support for end users, and monitoring of impact.^6^, ^17^ Legislation such as the Health Information Technology for Economic and Clinical Health (HITECH) Act and 21st Century Cures Act has supported point of care CDS, focusing primarily on interoperability requirements, but integration of genomic information needs to be addressed more explicitly. Important data management issues to be explored include responsibility for data governance and retention, variant reinterpretation, and managing secondary findings not ordered clinically, as well as funding for genomic data storage and analysis^12^ (Box 2).

**BOX 2:** Research topics that could be addressed by a network of gLHS.

Examine sustainability models beyond grant‐supported or institutional funding Develop innovative, low‐cost approaches to including under‐resourced settings in gLHS networks and collecting information needed to account for or minimize biases due to SDOH Identify and address potential limitations to sharing of genomic and associated clinical data by sharing diagnoses, variants, and de‐identified phenotypic information Investigate approaches for identifying and addressing licensing requirements for eConsults Explore and provide potential solutions to issues such as: Responsibility for data governance and retention Genomic variant reinterpretation Management of secondary findings Funding for genomic data storage and analysis

Early gLHS adopters, such as NorthShore's DNA10K program (now Endeavor Health), have engaged primary care clinicians in ordering genomic testing for high‐risk patients and have built end‐to‐end, shareable software to facilitate this workflow.^18^, ^19^ Vanderbilt's PREDICT program^6^ has advanced the application of available data standards to near‐seamless integration of pharmacogenomic variant information with clinical workflows. Data from multiple sources are aggregated in this platform and presented in summary form through a dashboard that enables queries for specific clinical or genomic characteristics. Relevant information is also automatically channeled to a patient‐facing portal providing lay interpretations and urging patients to discuss results with their clinicians.^6^ EHR companies can make genomic variant interpretations and clinical recommendations available through special applications, such as the genomic indicators module of Epic Systems Corporation (Verona WI).^5^, ^6^, ^7^ Examples of the international scope of such efforts are PhenoPackets^20^ and the Variant Representation Specification^21^; both are proposed international standards developed through the Global Alliance for Genomics and Health (of which the National Institutes of Health through NHGRI is a strategic partner). These standards are being incorporated into research activities sponsored by NHGRI and others and into resources such as ClinGen to make them available for clinical use.

Current gLHS have developed resources for supporting non‐geneticist clinicians such as the genomic medicine consultation or “eConsult” services at Indiana University,^22^ Massachusetts General/Brigham,^23^, ^24^ Vanderbilt, and the Department of Veterans Affairs.^25^ These services help clinicians with questions such as whether and how to order testing or referrals and how to act upon results. At present, such services are isolated within institutions but could be expanded to provide remote consultation through a broader, even national, genomic medicine board or consult service. This would avoid the need for each institution to develop its own consult procedures, independently fielding similar questions and providing similar responses. This inefficient, siloed approach is reminiscent of the dispersed and conflicting nature of genomic variant interpretation before the advent of the ClinVar database and Clinical Genome Resource (ClinGen),^26^ through which laboratories can now share and access variant classifications^27^ and the global scientific community can engage in standardized, expert gene and variant annotation and classification.^28^ A centralized service could facilitate similar cross‐institution interoperability and support, though sustainable funding models for it would need to be developed. A potential barrier to such a regional or national system is the question of whether licensure is needed for peer‐to‐peer consultation if those providing consultation are not providing direct patient care.^29^ Opinions vary on this question^30^ and some creative solutions have been developed in other fields such as pediatric mental health care.^31^

New educational programs to address increasing demands for genomic medicine services and the relative paucity of clinical geneticists and genetic counselors are beginning to train non‐geneticist physicians in genomic medicine. These include Mount Sinai's Genomic Medicine Track and the University of Miami's M.D./M.S. in Genomic Medicine. Educational efforts are also expanding for allied health professionals such as nurses, physicians' assistants, and even bachelor's level genetic assistants.^32^ In 2016, NHGRI initiated institutional training grants (“T32”) in genomic medicine *research* to train post‐doctoral researchers in identifying solutions and generating evidence to increase the adoption of genomics in clinical care. Subsequent NHGRI programs have supported curriculum development for medical students and entry‐level clinical and research assistants. Expansion and sharing materials from such programs can increase the availability of consultative resources and support for clinical care.

Implementing genomic testing into the clinical workflow has also enabled generation of clinical evidence of its downstream impact, such as additional tests or procedures, diagnoses, clinical outcomes, and costs.^33^ EHR‐based studies of individuals with and without genomic testing focusing on standardized outcomes of importance to payers can provide compelling evidence to support or refute more widespread use of testing. Surveys of patients and clinicians can complement EHR data to assess the full spectrum of utility of and interest in genomic testing.

Significant advances in data exchange and interoperability are exemplified by the “Genetic Cancer Risk Detector (GARDE)” program of the University of Utah Health.^34^ This novel platform includes algorithms to identify patients for genetic testing based on EHR data such as family history and uses automated chatbots for patient outreach, education, and access to testing. GARDE is EHR‐agnostic and open‐source rather than proprietary—both being critical advances over other platforms. It addresses interoperability and scalability challenges by leveraging emerging standards including Health Level 7 (HL7) Fast Healthcare Interoperability Resources (FHIR) and CDS Hooks. Sustainability of such systems beyond initial grant or institutional funding is a significant challenge, and various sustainability models could be explored by a gLHS network. The value of GARDE was recently demonstrated by its successful deployment in three large healthcare systems utilizing two different EHRs,^34^ and has recently been extended to a total of six systems. The effectiveness and scalability of a GARDE‐based intervention for genetic testing of hereditary cancer has been recently demonstrated in the BRIDGE trial.^35^

These early successes can be expanded through wider adoption of standardized genomic data elements,^36^ access procedures, and consent provisions by integrating genomic data into health information exchanges (HIEs). HIEs share individual‐level electronic health information among providers, across clinical facilities, and with patients to coordinate and improve clinical care.^37^ Current HIEs are state‐based, regional, or tied to an EHR platform (e.g., Epic's Care Everywhere) but implementing nationwide exchange of health information—including genomics—through initiatives such as the Assistant Secretary for Technology Policy/Office of the National Coordinator's Trusted Exchange Framework and Common Agreement (TEFCA) and Sync for Genes projects would extend the reach of HIEs, facilitate care, and provide opportunities for research. In addition, cloud‐based resources can support secure storage of genomic‐based data and leverage various artificial intelligence‐based tools to assist in analysis, but interoperability among various cloud‐based resources has been a challenge. Other potential limitations to sharing genomic data should be explored and addressed. Expansion to under‐resourced settings must also be a priority and accommodate current barriers to accessing genomic testing, expert consultation, and a range of electronic technologies.^4^ Standardized data elements and access procedures (such as those implemented by Carequality ^38^ and the US Core Data for Interoperability, USCDI) can also accelerate critical public health research, as demonstrated during the COVID‐19 pandemic.^39^

Increased adoption of genomic data standards and improved interoperability can also facilitate patient access and potential control of their genomic information.^4^ Giving the one constant in the clinical care continuum—the patient—ongoing access to their health information allows them to transport their genomic data to other healthcare providers as needed, putting them at the center (and in control) of their genomic healthcare. Some gLHS are exploring providing this type of access and transportability for genomic data, such as the Geisinger MyCode Community Health Initiative^40^ and its patient‐facing genomic test reports.^41^ This and similar programs are also expanding engagement efforts to involve a broad range of stakeholders including policymakers, health systems leaders, and payers.^42^ Early evidence of improved patient outcomes from gLHS programs without increasing healthcare costs has recently been reported.^43^, ^44^

## 4 CONCLUSIONS

gLHS have incorporated and benefited from recent advances in genomic data standardization, automated CDS, increased interoperability, and improved genomic technologies. They are, however, still few in number and operate largely independently, but provide opportunities for sharing data and methods with other gLHS through qualified genomic HIEs. Such HIEs have the potential for improving genomic variant classification, diagnostic accuracy, and patient care, as well as facilitating the research and evidence generation focused on standardized patient outcomes that can serve all of these ends.

gLHS could also work together to hasten the evolution of such systems, developing creative solutions for limited‐resource settings and achieving near “plug‐and‐play” applications for new adopters. Much of the genomic indicators application now available in commercial systems takes advantage of knowledge generated by NHGRI‐supported networks such as eMERGE, ClinGen, AnVIL, and CPIC, as well as community‐based data sharing through ClinVar. Many systems highlighted here have expressed willingness to share their methods and tools. Bringing together a network of gLHS, as is now beginning to be done under NHGRI leadership, could enable sharing across systems that, in turn, could promote convergence upon best practices and their dissemination (Box 2). Such a coalition could also work to improve transportability of genomic information across healthcare systems, facilitate research to assess the effectiveness of genomic medicine interventions, and potentially increase gLHS equity by expanding the number of functioning gLHS to improve genomic medicine research and clinical care for all communities.

## CONFLICT OF INTEREST STATEMENT

Noura Abul‐Husn is an employee and equity holder of 23andMe and serves on the scientific advisory board for Allelica. Adam Buchanan owns an equity stake in MeTree and You, Inc. Janina M. Jeff is employed by Illumina. Nancy Mendelsohn was an employee of Optum Health at the time this work was conducted. Travis Osterman receives research funding from GenomOncology and advises eHealth Technologies and MD Outlook. Daryl Pritchard is employed by the Personalized Medicine Coalition. Heidi Rehm receives research funding from Illumina and Microsoft and is a compensated member of the Genome Medical Scientific Advisory Board. Carol Bult, Rex Chisholm, Christopher Chute, Guilherme Del Fiol, Patricia Deverka, Gai Elhanan, Geoffrey Ginsburg, Eric Green, Susanne Haga, Rizwan Hamid, Carol Horowitz, Peter Hulick, Cynthia James, Gail Jarvik, Bruce Korf, Latrice Landry, Howard McLeod, Teri Manolio, George Mensah, Jahnavi Narula, Erin Ramos, Mary Relling, Renee Rider, Dan Roden, Robb Rowley, Casey Overby Taylor, Krystal Tsosie, Jason Vassy, Karriem Watson, Ken Wiley, and Marc Williams have no conflicts to declare.
